# AnnexinA6: a potential therapeutic target gene for extracellular matrix mineralization

**DOI:** 10.3389/fcell.2023.1201200

**Published:** 2023-09-04

**Authors:** Jie Yang, Tong Pei, Guanyue Su, Peiyan Duan, Xiaoheng Liu

**Affiliations:** Institute of Biomedical Engineering, West China School of Basic Medical Sciences and Forensic Medicine, Sichuan University, Chengdu, China

**Keywords:** AnnexinA6, Ca^2+^ regulation, matrix vesicles, extracellular matrix mineralization, osteogenesis, bone regeneration

## Abstract

The mineralization of the extracellular matrix (ECM) is an essential and crucial process for physiological bone formation and pathological calcification. The abnormal function of ECM mineralization contributes to the worldwide risk of developing mineralization-related diseases; for instance, vascular calcification is attributed to the hyperfunction of ECM mineralization, while osteoporosis is due to hypofunction. AnnexinA6 (AnxA6), a Ca^2+^-dependent phospholipid-binding protein, has been extensively reported as an essential target in mineralization-related diseases such as osteoporosis, osteoarthritis, atherosclerosis, osteosarcoma, and calcific aortic valve disease. To date, AnxA6, as the largest member of the Annexin family, has attracted much attention due to its significant contribution to matrix vesicles (MVs) production and release, MVs-ECM interaction, cytoplasmic Ca^2+^ influx, and maturation of hydroxyapatite, making it an essential target in ECM mineralization. In this review, we outlined the recent advancements in the role of AnxA6 in mineralization-related diseases and the potential mechanisms of AnxA6 under normal and mineralization-related pathological conditions. AnxA6 could promote ECM mineralization for bone regeneration in the manner described previously. Therefore, AnxA6 may be a potential osteogenic target for ECM mineralization.

## Introduction

The extracellular matrix (ECM) mineralization is an orchestrated and fine-tuned biological process during which inorganic minerals are produced and deposited in ECM (Hasegawa et al., 2022). The formation of hydroxyapatite (HA), the core element of inorganic minerals, depends on the combined effects of surrounding calcium ions (Ca^2+^), inorganic phosphate (PO_4_^3−^), as well as matrix vesicles (MVs) (Wuthier and Lipscomb, 2011; Bottini et al., 2018; Veschi et al., 2022). Annexins (Anxs) are Ca^2+^-dependent phospholipid-binding proteins that emerge as a multigene family with a conserved evolutionary origin and are widely distributed in animals and plants. Increasingly, investigations have shown that annexins regulate Ca^2+^ influx and intracellular Ca^2+^ concentrations (Gerke and Moss, 2002). Ca^2+^ and annexins are considered critical elements involved in mineralization and play central roles in the initiation and maturation of ECM mineralization (Balcerzak et al., 2003).

ECM mineralization involves the formation of calcium phosphate crystals from the combination of Ca^2+^ and PO_4_^3−^, which are then deposited on the collagen of the ECM. Briefly, membrane transporters and enzymes, including ATP-dependent Ca^2+^ pumps, and annexins as previously reported (Benz et al., 1996; Hoyal et al., 1996; Naciff et al., 1996; Fleet et al., 1999), initially transported Ca^2+^ and PO_4_^3−^ in extracellular fluid into MVs to form hydroxyapatite crystals. Afterward, the crystals gradually grow in the MVs, penetrate through the membrane of the MVs, deposit on collagen fibers, and ultimately form mineralization nodules. Of note, the levels and locations of ECM mineralization vary according to different physiological and pathological conditions. Physiological mineralization is typically observed in bone, tooth, and epiphyseal plates. In contrast, pathological mineralization known as “ectopic calcification” occurs in the arteries, myocardium, joints, and brain, leading to atherosclerosis, osteoarthritis, and calcific aortic valve disease (Maré et al.t, 2020), and some genetic diseases (Hahn et al., 2015; Reiss et al., 2018; Sherwood, 2019). Therefore, it is of great clinical significance to investigate the regulatory mechanisms of ECM mineralization and uncover the potential therapeutic targets for related diseases.

AnnexinA6 (AnxA6), a major component of MVs, has been reported to regulate physiological ECM mineralization by participating in Ca^2+^ transport and forming mineralized nucleation sites (Davies et al., 2019; Veschi et al., 2020; Veschi et al., 2022). AnxA6 has a Ca^2+^ transport capacity that can mediate Ca^2+^ influx into artificial liposomes (Matsuda et al., 1997). Some evidence suggests that AnxA6 may have Ca^2+^ channel properties, which mediate Ca^2+^ influx into MVs (Kirsch et al., 2000). AnxA6 forms voltage-dependent Ca^2+^ channels when inserted into artificial phosphatidylserine bilayers (Benz et al., 1996). However, deterministic conclusions still need to be justified by many studies. Given the vital role of AnxA6 in ECM mineralization and osteogenesis (Bolean et al., 2020; Bozycki et al., 2021; Mroczek et al., 2022; Pei et al., 2022), we reviewed the biological function of AnxA6 in regulating physiological and pathological ECM mineralization. We illustrated the underlying mechanisms to provide novel perspectives for mineralization-associated studies and contribute to treating mineralization-related diseases.

## The structure and biological functions of AnxA6

Annexin is a group of Ca^2+^-dependent multifunctional lipid-binding proteins that Creutz discovered in 1978 (Creutz et al., 1978). According to the expression in different species, Anx is generally categorized into five groups: A (vertebrates), B (invertebrates), C (in fungi and some groups of unicellular eukaryotes), D (plants), and E (protists) (Moss and Morgan, 2004). In vertebrates, 12 kinds of AnxA members, including A1-A11 and A13, have been found. AnxAs have been reported to exert diverse functions in human systems and play different roles in regulating the progression of many diseases. The functions and distributions of AnxAs and AnxAs-associated diseases in humans are highlighted and summarized in Table 1.

**TABLE 1:** Properties and potential cellular functions of mammalian AnxAs.

Annexin	Gene encoding	Total aa	Function	Distribution	Diseases	References
AnnexinxA1	ANXA1	346	Inflammatory response, Wound healing, Cancer cell metastasis, hormone secretion, Vesicle fusion, Signal transduction, Viral uptake, Apoptosis, T-cell activation, Phagocytosis	In most tissues and cells	Breast cancer, Hormone-refractory prostate cancer, Diabetic nephropathy, Cerebral ischemia-reperfusion injury, Stroke, Neurodegenerative condition, Periprosthetic bone loss	Chen et al. (2021), Foo et al. (2019), Leoni et al. (2015), Li et al. (2021), McArthur et al. (2020), Onuora (2022), Perretti & D'Acquisto (2009), Solito et al. (2008), Wu et al. (2021), Xu et al. (2021)
				Abundant expression in differentiated cells		
AnnexinA2	ANXA2	357	Vesicle fusion, Antithrombotic, Cancer cell metastasis, Fibrinolysis, Defense against bacterial infection, Activate osteoclasts, angiogenesis, Plasma membrane repair, Cholesterol transport, Autophagy, Macrophage phenotypic change	In most tissues and cells	Atherosclerosis, Heart failure, Acute promyelocytic leukemia, Breast cancer, Diabetes, Pulmonary fibrosis, Prostate cancer, Preeclampsia, kidney diseases	Li et al. (2005), Madureira et al. (2011), Cañas et al. (2015), Demonbreun et al. (2016), Grewal et al. (2016), Lin and Hu, 2017 (2022), Wang et al. (2018), Foltz et al. (2021), Tan et al. (2021), Garrido-Gómez et al. (2022)
		339		Abundant expression in the pancreas, colon, ileum, and adrenal gland		
AnnexinA3	ANXA3	323	Autophagy, Apoptosis, Cancer cell metastasis, Signal transduction	In most tissues and cells	Breast cancer, acute myocardial infarction, Pain, Hepatocellular carcinoma, Ankylosing spondylitis, Intracranial aneurysm, Pancreatic ductal adenocarcinoma	Du et al. (2018), Tong et al. (2018), Meng et al. (2019), Wang, Wang, Yang and Cheng (2019), Wan et al. (2020), Zhang et al. (2020), Guo et al. (2021), Yang et al. (2021), Jiang J et al. (2022), Liang et al. (2023)
				Abundant expression in skeleton		
AnnexinA4	ANXA4	321	Vesicle fusion, Signal transduction, Apoptosis, Inflammatory response, Plasma membrane repair, Anti-coagulant	In most tissues and cells	Gastric cancer, Renal cell carcinoma, Preeclampsia, Glaucoma, Oral squamous cell carcinoma	Wei et al., 2015; Boye et al. (2017), Xu et al. (2019), Nakayama et al. (2020), Croissant et al. (2022), Vicic et al. (2022), Zhang et al. (2023)
				Abundant expression in the gallbladder, pancreas		
AnnexinA5	ANXA5	320	Phagocytosis, Biomineralization, Thrombosis, Angiogenesis, Recognition of apoptotic cells, Cancer diagnosis, Anti-coagulant, Signal transduction	In most tissues and cells	Heart failure, Prostate cancer, Leukemia, Myocardial infarction, Cutaneous squamous cell carcinoma, Recurrent miscarriage, bone growth	Garnier et al. (2009), Bouter et al. (2011), Peng et al. (2014), Bouter et al. (2015), Ormesher and Greer (2016), Shimada et al. (2018), Kang et al. (2020), Woodward et al. (2022), Gao et al. (2023)
				Not expressed in neuronal cells		
AnnexinA6	ANXA6	673	Signal transduction, Calcium ion homeostasis, Plasma membrane repair, Muscle contraction, Gluconeogenesis, Biomineralization, Chondrocyte differentiation, Apoptosis	In most tissues and cells	Myositis, Heart Failure, Melanoma, Hormone-Refractory Prostate Cancer	Buzhynskyy et al. (2009), Swaggart et al. (2014), Middel et al. (2016), Boye et al. (2017), Demonbreun et al. (2019), Croissant et al. (2020), Demonbreun et al. (2022), Gounou et al. (2023)
		641		Abundant expression in skeletal, skeletal muscle, liver, heart, and lymph nodes		
AnnexinA7	ANXA7	488	Vesicle fusion, Autophagy, Tumor suppressor, Cardiac contraction and reconstitution, Insulin excretion, Cell proliferation, Apoptosis	In most tissues and cells	Prostate cancer, Recurrent pregnancy loss	Gerke and Moss (2002), Liu et al. (2018), Schloer et al., 2018; Lin et al. (2019), Alauddin et al. (2020), Meng et al. (2020), Manke et al. (2021), Chen et al. (2023)
		466		Isoform 1 is highly expressed in the human brain, heart, and skeletal muscle. Isoform 2 is more plentiful in the placenta, kidney, spleen, lung, fibroblasts, and liver		
AnnexinA8	ANXA8	327	Endosomal transport, Anti-coagulant, Angiogenesis	In most tissues and cells	Ovarian cancer, Age-related macular degeneration	Heitzig et al. (2017), Heitzig et al. (2018), Gou et al. (2019), Lueck et al. (2020), Ma et al. (2020)
				Abundantly expressed in the esophagus, skin, vagina		
AnnexinA9	ANXA9	345	Cell adhesion, Cancer cell metastasis	In most tissues and cells	Lung adenocarcinoma, Gastric cancer, Colorectal cancer	Salom et al. (2019), Zhou et al. (2021), Lu et al. (2023), Wang et al. (2023)
AnnexinA10	ANXA10	324	Apoptosis, Signal transduction	In most tissues and cells	Intrahepatic cholangiocarcinoma, Pancreatic ductal adenocarcinoma, Gastric adenocarcinoma, Papillary thyroid cancer	Sun et al. (2019), Liu et al. (2021), Wei and Zhu (2021), Ishikawa et al. (2022a), Ishikawa et al. (2022b), Shao et al. (2022)
				Abundantly expressed in the stomach		
AnnexinA11	ANXA11	505	Phagocytosis, Ca^2+^ transduction, Lysosome, calcium homeostasis	In most tissues and cells	Amyotrophic lateral sclerosis, Gastric cancer	Smith et al. (2017), Hua et al. (2018), Liao et al. (2019), Lillebostad et al. (2020), Nahm et al. (2020), Jiang Q et al. (2022), Johari et al. (2022)
AnnexinA13	ANXA13	316	Exocytosis, Cell differentiation, Membrane fusion	Specifically expressed in epithelial cells of the colon and jejunum	Acute promyelocytic leukemia, Lung adenocarcinoma	Filipenko et al. (2004), McCulloch et al. (2019), Xue et al. (2020)
		357				

Information is taken from https://www.uniprot.org.

**FIGURE 1:**
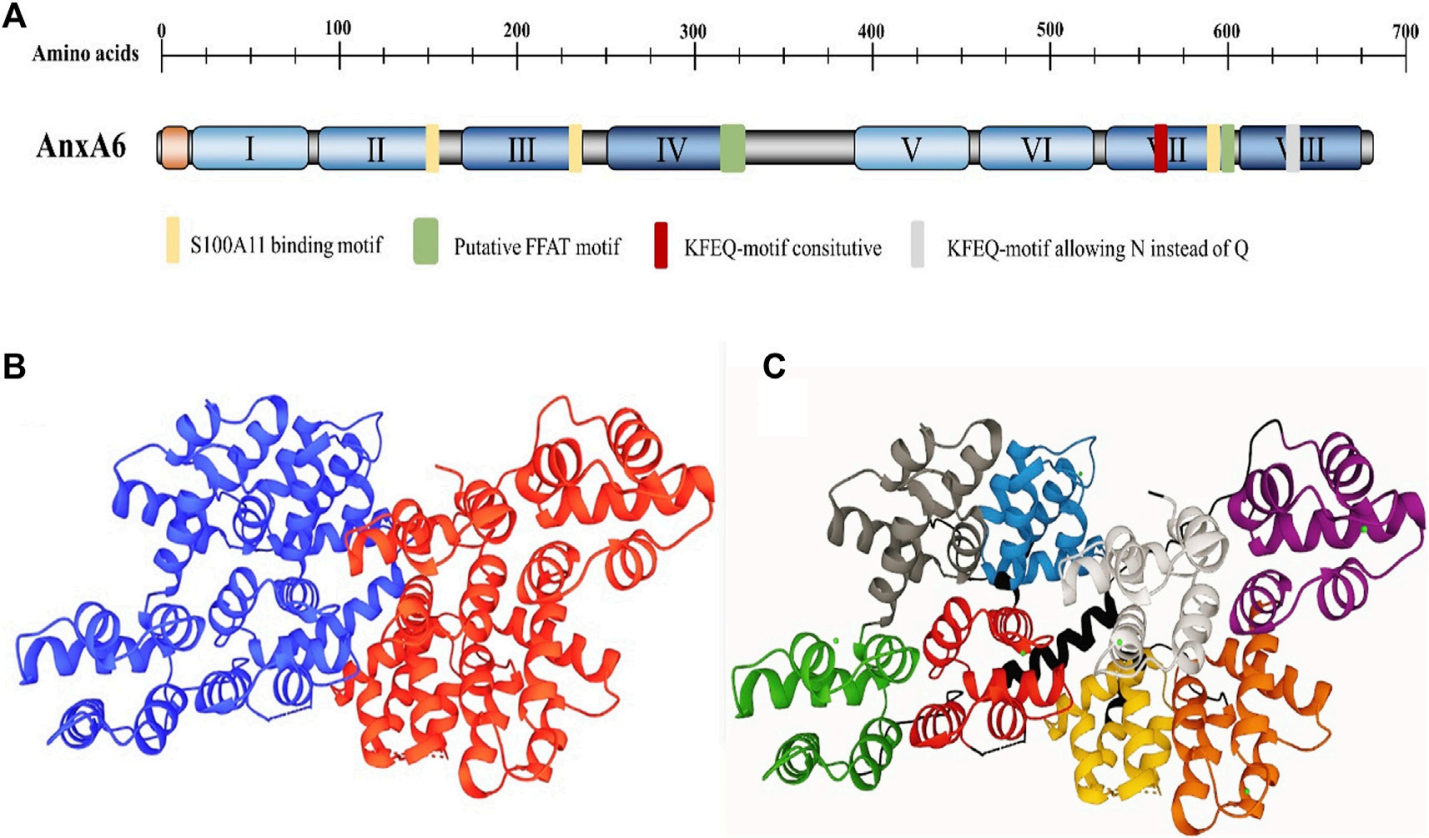
The structural organization of AnxA6. **(A)** Domain structure of AnxA6. The domain structure of AnxA6 is indicated. The N-terminal head domain (orange), C-terminal core with the AnxA6 repeats I–VIII (the only member within the Anx family), and length (in amino acids) are clarified. **(B)** The structure of AnxA6. AnxA6 consists of two lobes and a linker. The lobes are indicated by A (blue) and B (red). **(C)** Each lobe comprises four domains. The domain description and residue correspondence are as follows: Lobe A: N-terminal tail (1-19); domain I (green, 20-91); domain II (gray, 92-163); domain III (blue, 175-247); domain IV (red, 251-322). Interlobe linker: (black, 323-362). Lobe B: domain V (purple, 363-434); Domain VI (brown, 435-506); Domain VII (yellow, 521-595); Domain VIII (white, 599-670) (PDB file 1AVC).

As the largest member of the AnxAs family, the molecular weight (MW) of AnxA6 is up to 68 kDa (Huber et al., 1990a). In contrast to other AnxAs family proteins with only four homeodomains (Huber et al., 1990b), AnxA6 has a highly conserved core containing eight homeodomains, which further influences the function of AnxA6, such as membrane crosslinking and fold stabilization at high Ca^2+^ (Boye et al., 2018) (Figure 1). AnxA6 consists of two domains connected by hinge-like loops, which are not found in other AnxAs (Cornely et al., 2011). The Ca^2+^ binding sites of AnxA6 are positioned in repeats 1, 2, 4, 5, 6, and 8 (Huber et al., 1990a). The repeated domains may be derived from the evolution of integrating repeats of the AnxA5 and AnxA10 genes (Enrich et al., 2011). Alternative splicing of the AnxA6 gene produces two varying isoforms, AnxA6-1 and AnxA6-2, with a similar molecular weight of approximately 35 kDa (Avila-Sakar et al., 1998). AnxA6-1, found in most mammalian tissues, shows higher hydrophobicity and negative surface charges, while AnxA6-2, discovered in some immortalized cell lines (González-Noriega et al., 2016), is more affinitive to Ca^2+^ (Cornely et al., 2011). As reported, AnxA6 plays a biological role in mediating membrane receptor binding (Cornely et al., 2011), endocellular transport (Rentero et al., 2018), cytoskeleton reconstitution (Alvarez-Guaita et al., 2015), and transportation processes (Alvarez-Guaita et al., 2020), as well as being involved in physiological or pathological processes that are closely associated with the advancement of various diseases. The roles of AnxA6 in diseases are shown in Table 2.

**TABLE 2:** Roles of AnnexinA6 in varieties of diseases.

Diseases	AnxA6 expression schema	Implication	References
Melanoma	Low expression levels in melanoma malignancy	Suppressor	Trilla-Fuertes et al. (2019), Nguyen et al. (2022)
Epithelial carcinoma	No expression in A431 cells	Suppressor	Hoque et al. (2020), Jose et al. (2022)
Breast cancer	Low expression levels in breast cancer	Potential marker for detection	Korolkova et al. (2020)
Gastric cancer	Low expression levels in gastric cancer	Suppressor	Zhao et al. (2022)
Prostate cancer	Low expression levels in prostate cancer	Potential activator	Clark et al. (2023)
Chronic myeloid leukemia	Low expression levels in chronic myeloid leukemia	Suppressor	Qi et al. (2015)
Acute myeloid leukemia	High expression levels in acute myeloid leukemia	Promotor	Niu et al. (2019)
Cervical cancer	High expression levels in cervical carcinoma	Potential marker of diagnostics and prognosis	Sun et al. (2020)
Lymphoblastic leukemia	High expression levels in lymphoblastic leukemia	Potential marker for monitoring	Smith et al. (2002)
Heart failure	High expression levels in heart failure	Promotor	Demonbreun et al. (2022)
Psoriasis	A susceptibility factor	A susceptibility factor	Yan et al. (2022)
Diabetes	An associated gene	Promotor	Stogbauer et al. (2009)
Muscular dystrophies	A genetic modifier	Suppressor	(Croissant et al., 2021)

However, despite these good insights, the need for an apparent phenotype in AnxA6 KO mice (Hawkins et al., 1999) has led to some questions about the role of AnxA6 in mineralization. AnxA6 is highly expressed in the skeleton, but no abnormalities of skeletal development have been found in AnxA6 KO mice or even in the double KO mice of AnxA6 and AnxA5 (Belluoccio et al., 2010; Grskovic et al., 2012). Still, Subsequent analysis of the skeletal phenotype of AnxA6 KO newborns shows a reduction in growth plate length and chondrocyte number, possibly due to reduced cartilage mineralization in the growth plate (Minashima et al., 2012). In addition, primary chondrocytes derived from AnxA6 KO mice show delayed terminal differentiation and reduced PKCα membrane translocation and activity, which may be one of the reasons for reduced MAPK signaling in chondrocytes (Minashima et al., 2012). In a study on osteoarthritis, cartilage destruction in knee joints was significantly reduced in AnxA6 KO mice, possibly due to reduced NFκB activity (Campbell et al., 2013). In contrast, in articular chondrocytes from control animals, AnxA6 attenuated cartilage degradation by interfering with the crosstalk between the Wnt/b-catenin signaling pathway and NFκB signaling, reducing catabolism, metabolism, and inflammatory responses in knee cartilage (Minashima and Kirsch, 2018). These studies on AnxA6 KO mice highlight the great therapeutic value of AnxA6 and the feasibility of AnxA6 for studying mineralization-related diseases.

## AnxA6 participates in mineralization-related diseases

### Osteoporosis (OP)

Osteoporosis is a common metabolic bone disease characterized by enhanced bone turnover, decreased bone mass, and susceptibility to fracture (Aibar-Almazán et al., 2022). Bone remodeling is a dynamic process during which the bone constantly experiences destruction and replacement. OP exists when the formation of new bone doesn’t follow the reduction of old bone. A case-control analysis in the Korean Women’s Cohort (3,570 subjects) has indicated that genetic variation of AnxA6 is significantly associated with OP (kim, 2011). The possible reasons are as follows: First, AnxA6 regulates osteoblast proliferation, differentiation, necrosis, and apoptosis (Kim et al., 2011); second, AnxA6 may interact with either phospholipids or type I collagen to induce the nucleation process in MVs-mediated mineralization (Veschi et al., 2020; Veschi et al., 2022). Accordingly, AnxA6 enhances the occurrence and progression of OP by exerting a significant influence on osteoblasts.

### Osteoarthritis (OA)

Osteoarthritis is the most common joint disease, with more than 240 million people at risk worldwide (Katz et al., 2021). OA is generally represented by cartilage degeneration, bone remodeling, osteophyte generation, and joint dysfunction (Kraus et al., 2015). AnxA6 is reported to closely link with matrix vesicle-mediated mineralization of growth plate cartilage (Kirsch et al., 2000; Pfander et al., 2001). Notably, AnxA6 can be regarded as a marker in human osteoarthritic chondrocytes due to its high expression in OA cartilage, whereas low expression in healthy articular cartilage (Minashima et al., 2013). AnxA6 has been identified as a mediator of Ca^2+^ influx across membranes, leading to the induction of mineralization events in OA (Minashima et al., 2012). To test whether AnxA6 forms a Ca^2+^ channel in the plasma membrane, chondrocytes were treated with retinoic acid (RA) and antibodies specific to AnxA6. The anti–AnxA6 IgG fraction decreased the RA-mediated increase in the cytosolic calcium concentration by 65%, indicating that AnxA6 in the plasma membrane of growth plate chondrocytes and mediates Ca^2+^ influx (Wang and Kirsch, 2002). The underlying mechanisms of AnxA6-driven mineralization in OA probably depend on NF-κB and Wnt/β-catenin signaling pathways and their cross-talk (T. Minashima and Kirsch, 2018).

### Atherosclerosis (AS)

Atherosclerosis is a chronic and complex inflammatory disease that can lead to life-threatening events, concentrated in most deaths worldwide (Libby, 2021). It is known that nidus calcification in atherosclerosis is widespread and enhanced with age (Hoffmann et al., 2003). Coronary artery calcium score, a measure of the total amount of calcification, is a positive biomarker of coronary plaque burden and offers prognostic information beyond that gained by conventional risk factor scoring (Pletcher et al., 2004). AnxA6 plays an essential role in the pathological calcification process of atherosclerosis, in which mature contractile vascular smooth muscle cells (VSMCs) withstand phenotypic transitions in response to pathological factors such as aging, oxidative stress, inflammation, and mechanical injury, leading to vascular ECM calcification (Gomez and Owens, 2012). Studies have revealed that vascular calcification is a strictly modulated process similar to bone mineralization (Shanahan et al., 2011). MVs-mediated mineralization is the primary pathological process that AnxA6 may participate in Reynolds et al. (2004) and New et al. (2013). That is to say, calcifying factors induce the secretion of MVs characterized by increased phosphatidyl serine and AnxA6 content, subsequently leading to vascular ECM calcification (Liberman and Marti, 2017). Meanwhile, an interesting study showed that AnxA6 was enriched in MVs derived from osteogenic medium-cultured smooth muscle cells (Rogers et al., 2020). Besides, several studies indicated that MVs from calcified smooth muscle cells had an increased AnxA6 content (Chen et al., 2008), as AnxA6 was also abundant at sites of vascular calcification *in vivo* (Kapustin et al., 2011).

### Osteosarcoma (OS)

Osteosarcoma is the most common primary bone malignancy (Yang et al., 2020; Shoaib et al., 2022), characterized by osteolytic lesions radiographically (Kansara et al., 2014). A mineralized microenvironment is reported to induce osteogenic differentiation of mesenchymal stem cells, thus reducing OS progression (Rubio et al., 2014). Stimulation of cells for mineralization resulted in an upregulation of AnxA6 expression in OS Saos-2 cells, whereas its expression level significantly decreased upon inhibition of calcium channel activity. The existing evidence suggests that the membranous co-localization of AnxA6 and TNAP enhances submembrane mineralization (Bozycki et al., 2021). Additionally, AnxA6 is recruited to the membrane by co-localizing with cofilin-1 during MVs formation and participates in the mineralization process of OS Saos-2 cells (Thouverey et al., 2009). This approach can serve as a novel therapeutic intervention for osteoporosis by facilitating the process of mineralization.

### Calcific aortic valve disease (CAVD)

**FIGURE 2:**
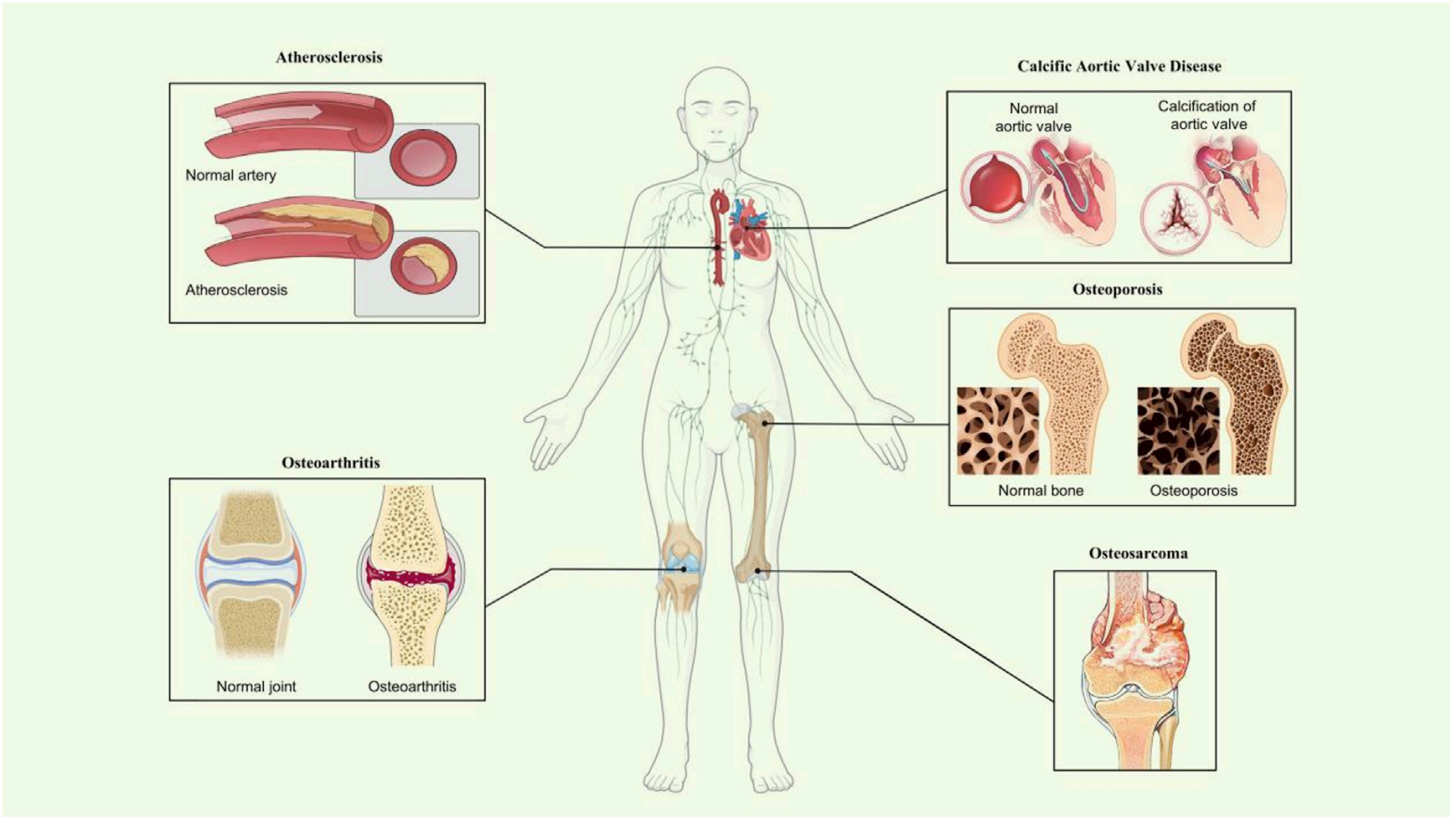
Roles of AnxA6 in mineralization-related disease. Schematic diagram showing what is known about AnxA6 in osteoporosis and osteoarthritis, atherosclerosis, osteosarcoma, and calcific aortic valve disease of mineralization-related diseases.

Calcific aortic valve disease is a highly prevalent heart valve disease globally (Kraler et al., 2022). The pathophysiology of CAVD is complicated and influenced by various factors such as mechanical stress (Zhong et al., 2023), genetic factors (Iqbal et al., 2023), and inflammation (Broeders et al., 2022), but it shares similar mechanisms with physiological bone formation (Gollmann−Tepeköylü et al., 2023). The valvular interstitial cells (VICs) are the most plentiful type in the aortic valve and play a crucial role in CAVD development (Wu et al., 2017). VICs can transform into osteoblast-like cells, which cause osteogenic differentiation and calcification, consequently leading to the onset of CAVD. Previous research showed that 4-Octyl itaconate alleviated CAVD by ameliorating the osteogenic response of VICs (Peng et al., 2022). In addition, miR-22, as a promotor of the osteogenic differentiation of VICs, accelerated the process of CAVD (Yang et al., 2022). VIC-derived MVs (Cui et al., 2017) are critical in CAVD. Accordingly, AnxA6 was remarkably upregulated in calcified VIC-derived MVs in the calcified aortic valve compared with normal VICs. These data demonstrate the possible role of AnxA6 in the development of CAVD (Figure 2).

## How does AnxA6 work during the physiological mineralization process?

### AnxA6 promotes MVs generation and release

AnxA6 plays a crucial role in the generation and release of MVs. Studies have demonstrated that AnxA6 is enriched in MVs secreted by osteoblasts and hypertrophic chondrocytes in bone formation (Minashima et al., 2012). Previous studies indicated that elevated AnxA6 positively promoted the release of mineralization-competent MVs (Bozycki et al., 2021). The formation of MVs may be associated with the processes concerning AnxAs uncoupling from the cytoskeletal network (Wuthier and Lipscomb, 2011). Over the years, emerging evidence has shown that AnxA6 is involved in the formation of extracellular vesicles by mediating the fusion of early endosomes (Enrich et al., 2017), autophagosome/lysosome (Ghislat and Knecht, 2012), and late endosomes (LE)/lysosome (Futter and White, 2007). MVs are generally recognized to release from the cells by membrane budding, with complex regulatory mechanisms. Serving as an intracellular MVs biogenesis pathway, the effect of the mitochondria-lysosome axis has been previously identified (Iwayama et al., 2022). To date, AnxA6 has been reported to mediate the reprogramming of membrane-cytoskeleton interactions to upgrade membrane curvature, an initial condition for vesicle budding (Rentero et al., 2018). Furthermore, surface AnxA6 on the cell membrane interacts with proteins such as spectrin and dynamin, required for clathrin-coated vesicle budding and endocytic vesicle stripping of the plasma membrane (Grewal et al., 2000). AnxA6 is believed to be a key element in cell membrane fusion and budding events, which are essential for MVs generation and release.

### AnxA6 promotes MVs binding to the ECM

After MVs are generated and secreted into the extracellular space, they will tightly anchor to the collagen in the ECM and initiate the secondary mineralization stage. The interaction between MVs and ECM mutually affected the extracellular accumulation and aggregation of calcified MVs (T.Li et al., 2022). Osteoblasts are generally responsible for synthesizing type I collagen-rich ECM, which is necessary for osteogenic mineralization (Bottini et al., 2018). In general, the linking role of AnxA6 should be analyzed from two aspects, including type I collagen and MVs membranes. Recently, AnxA6-loaded liposomes have been used to explore the role of AnxA6 in MVs-mediated mineralization, and the findings suggest that AnxA6 may exert its nucleation and mineralization abilities by necessary anchoring to type I collagen (Veschi et al., 2022). Other studies have reported similar findings (Chen et al., 2008). Take vascular calcification as an example; AnxA6 is enriched in calcified MVs and interacts with type I collagen to promote the mineralization processes (Kirsch et al., 2000). On the MVs membrane side, AnxA6 interacts with membranes by a lipid-related mechanism. In addition to perturbing cholesterol distribution (Swaggart et al., 2014), AnxA6 can bind to phosphatidylcholine on the MVs surface, which may contribute significantly to the interaction between MVs and collagen fibrils (Veschi et al., 2020).

### AnxA6 promotes calcium influx in cells and MVs

As is known, the levels of Ca^2+^ are a critical determinant for ECM mineralization (Murshed, 2018). AnxA6 has been proposed to facilitate the influx of Ca2+ into mineralized MVs, as previously mentioned (Benz et al., 1996; Kirsch et al., 2000), possibly depending on two specific functional domains: 1) Ca2+ and lipid binding domains (Montaville et al., 2002), which are responsible for Ca2+ transport to endosomes by binding AnxA6 to cholesterol (de Diego et al., 2002); 2) pH-sensitive domains, which regulate the ion channel activity by affecting the folding degree of AnxA6 under different pH conditions (Golczak et al., 2001a; Golczak et a;., 2001b) and providing the foundation for Ca^2+^ influx.

Two isoforms of AnxA6, AnxA6-1 and AnxA6-2, exert different functions for Ca^2+^ influx due to their different structures. Existing data suggest that AnxA6-2 has a greater affinity for Ca^2+^ (Kaetzel et al., 1994). AnxA6-2 can form a narrower region with better Ca^2+^ binding ability. Furthermore, AnxA6-2 has a more comprehensive pH response range and is sensitive to changes in Ca^2+^ and proton concentration (Strzelecka−Kiliszek et al., 2008). In addition to the two isoforms above, a 35-kDa fragment of AnxA6 is also present in MVs (Wu et al., 1993), which is responsive to collagenase and/or endogenous proteases (Mookhtiar and Van Wart, 1992; Chen and Golub, 2001) and can tightly bind to calcium ions. AnxA6 promotes Ca^2+^ influx due to its location in the outer lobe of bilayer structures (Kirsch et al., 2000). Besides its Ca^2+^ channel activity in cells such as osteogenic differentiated chondrocytes, AnxA6 also plays a crucial role in Ca^2+^ influx in MVs (Kirsch et al., 2000). AnxA6 was identified to regulate mineralization events of chondrocytes by interacting with Protein Kinase C (PKC) and subsequently regulate Ca^2+^ influx in MVs (Minashima et al., 2012). AnxA6 knockdown, on the other hand, inhibited chondrocyte terminal differentiation and calcium uptake capacity (Grewal et al., 2016), preventing internal Ca^2+^ influx in both cells and MVs (Minashima et al., 2012). In conclusion, AnxA6 likely promotes ECM mineralization by facilitating the influx of Ca^2+^ into mineralized MVs.

### AnxA6 promotes nucleation core formation in MVs

AnxA6 is a major content protein of MVs and can also form nucleation sites upon binding to the MVs (Genge et al., 2007). SDS-PAGE characterization, Fourier-transform infrared, and NMR (Sauer and Wuthier, 1988; Genge et al., 1989; Genge et al., 1990; Wu et al., 1997) indicate that there are three crucial components in the nucleation core: 1) amorphous calcium phosphate (ACP); 2) phosphatidylserine-Ca2+-Pi complexes (PS-CPLX); 3) AnxAs, including AnxA6 (Wu et al., 1997).

**FIGURE 3:**
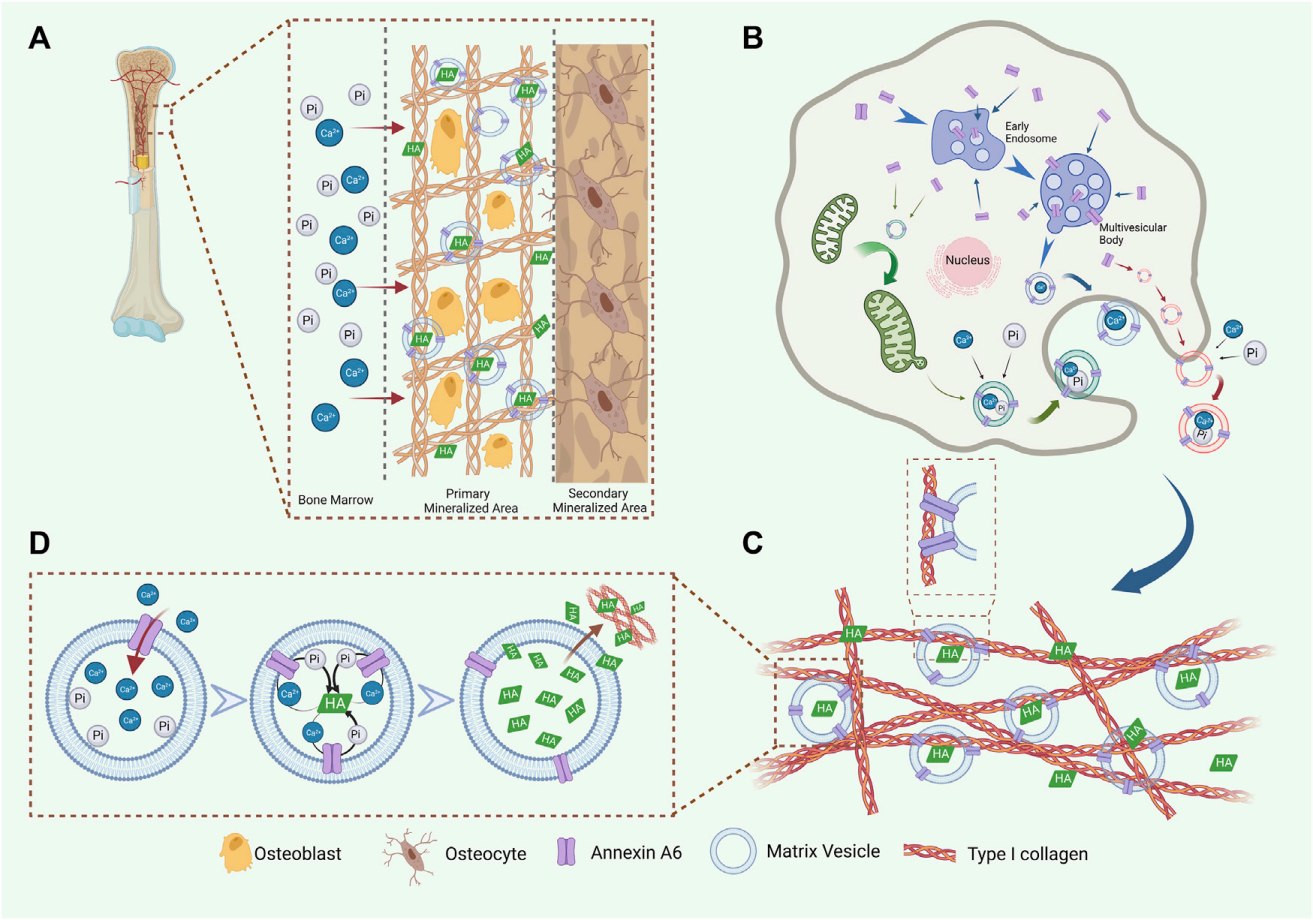
AnxA6 is involved in the formation, release, and Ca^2+^ influx of MVs. **(A)** A schematic representation of AnxA6 in ECM mineralization. **(B)** AnxA6 likely enhances the formation of MVs via three cooperative/redundant mechanisms include (i) MVs (red), which accumulate calcium (Ca^2+^) and phosphate (PO_4_^3−^) ions extracellularly, bud from the plasma membrane; (ii) MVs (green), which transport amorphous calcium phosphate and ionic calcium stored in mitochondria to the ECM; (iii) MVs (blue), originated from multivesicular bodies (MVBs) in “exosome-like” biogenesis pathway. **(C)** AnxA6 mediates MVs’ tight binding to type I collagen in the ECM, which benefits ECM mineralization. **(D)** AnxA6 on the MVs membrane facilitates Ca^2+^ influx and the formation of HA within MVs. Subsequently, AnxA6 drains HA outside to promote ECM mineralization.

How does AnxA6 contribute to nucleation core formation? First, the conformational variants of AnxA6 (Avila−Sakar et al., 2000) facilitate the nucleation of crystalline Ca-Pi. Second, AnxA6 promotes nucleation core formation due to its unique ability to bind to sphingolipids and cholesterol, which are abundant in membrane rafts (Babiychuk et al., 1999; Babiychuk and Draeger, 2000). Third, AnxA6 can further promote the accumulation of Ca^2+^ and stabilize the combination of Ca^2+^ and PS (Veschi et al., 2022), thus leading to hydroxyapatite formation. Finally, AnxA6 transfers from the inner surface of MVs to the outer surface and binds to phosphatidylcholine (PC) on the outer surface of MVs (Figure 3).

## Conclusion and perspectives

In conclusion, AnxA6 has novel biological functions and potential therapeutic applications in the mineralization of extracellular matrix, which may provide promising AnxA6-based therapeutic strategies for mineralization-related diseases, pave a novel way for drug discovery, and pursue AnxA6-based therapeutic strategies for mineralization-related diseases. AnxA6 can create an enabling environment for hydroxyapatite formation by promoting Ca^2+^ influx. Additionally, as an essential component of MVs, AnxA6 promotes the attachment of MVs to ECM. However, there remain questions that need to be further addressed. AnxA6 has two isoforms, but the significance of these isoforms in mineralization-related progression has yet to be thoroughly investigated. Further studies are needed to better understand the interactions among AnxA6 subtypes and clarify the mechanisms of AnxA6-promoted ECM mineralization. A better understanding of these mechanisms may contribute to developing mineralization-related disease therapies. Moreover, to move such research forward, the translational capacity of AnxA6 should be confirmed through clinical trials in the future.
